# Influence of Baseline Disease Severity on Rehabilitation Outcomes Following a Structured Exercise Program in Individuals With Chronic Parkinson’s Disease: A Secondary Analysis

**DOI:** 10.7759/cureus.114631

**Published:** 2026-08-16

**Authors:** Mukta T Killedar, Suraj B Kanase, Sakshi P Kavitake

**Affiliations:** 1 Department of Neurosciences Physiotherapy, Krishna Vishwa Vidyapeeth (Deemed to be University), Karad, IND

**Keywords:** disease severity, exercise rehabilitation, functional mobility, modified hoehn and yahr stage, parkinson's disease, physiotherapy, secondary analysis

## Abstract

Introduction

Parkinson's disease is a progressive neurodegenerative disorder characterized by impaired mobility, balance, and functional independence. Although structured exercise-based rehabilitation is beneficial, rehabilitation responsiveness varies among individuals. Baseline disease severity may influence functional recovery; however, evidence regarding its relationship with rehabilitation outcomes remains limited. This secondary analysis investigated the influence of baseline disease severity on rehabilitation outcomes following a structured physiotherapy rehabilitation program in individuals with chronic Parkinson's disease.

Methods

This secondary analysis used data from a previously completed single-group pretest-posttest quasi-experimental study involving 20 individuals with chronic Parkinson's disease (10 male patients and 10 female patients; age range: 50-70 years). Baseline disease severity was classified using the Modified Hoehn and Yahr stage. Functional mobility and balance were assessed using the Timed Up and Go (TUG) test and Berg Balance Scale (BBS), respectively. Spearman's rank correlation coefficient was used to evaluate the relationship between baseline disease severity and rehabilitation-related improvements, while changes in Modified Hoehn and Yahr stage were analyzed using the Wilcoxon signed-rank test.

Results

A statistically significant moderate positive correlation was observed between baseline Modified Hoehn and Yahr stage and improvement in TUG performance (rs=0.53, p=0.015). A weak positive correlation was found between baseline disease severity and improvement in BBS scores (rs=0.30, p=0.207), although this was not statistically significant. The median Modified Hoehn and Yahr stage decreased significantly from 2.75 (IQR: 2.50-3.25) to 2.00 (IQR: 2.00-2.63) following rehabilitation (p<0.001).

Conclusions

Baseline disease severity may influence rehabilitation outcomes in individuals with chronic Parkinson's disease. Greater baseline Modified Hoehn and Yahr stage was significantly associated with greater improvements in functional mobility, whereas balance-related improvements were observed across disease stages without a significant association with baseline disease severity. These findings suggest that individuals with more advanced baseline disease severity may still achieve meaningful improvements in functional mobility and that advanced disease should not automatically preclude participation in structured rehabilitation programs. Larger prospective studies are needed to confirm these observations.

## Introduction

Parkinson's disease (PD) is a chronic progressive neurodegenerative disorder and one of the leading causes of neurological disability among older adults worldwide [1-3]. The disease primarily results from degeneration of dopaminergic neurons within the substantia nigra, leading to disruption of basal ganglia circuits responsible for the regulation of voluntary movement [2]. Clinically, PD is characterized by bradykinesia, rigidity, resting tremor, and postural instability, all of which progressively impair gait, balance, mobility, and functional independence. In addition to motor manifestations, individuals frequently experience non-motor symptoms such as cognitive impairment, mood disturbances, sleep disorders, and autonomic dysfunction, which further contribute to reduced quality of life and increased disability [1].

The global prevalence of PD has increased substantially over recent decades, creating a considerable healthcare and socioeconomic burden worldwide [3]. As the disease progresses, individuals often experience increasing difficulty performing activities of daily living, reduced participation in community and social activities, and a greater risk of falls and fall-related injuries. Progressive deterioration in functional mobility and postural control further contributes to dependence and reduced overall well-being. Consequently, identifying effective long-term management strategies has become a major priority in the care of individuals with PD [3,4].

Although pharmacological therapy remains the cornerstone of treatment for motor symptoms, medication alone is often insufficient to address impairments related to balance, mobility, postural stability, and overall physical function [1]. Furthermore, long-term pharmacological treatment may be associated with complications such as motor fluctuations and dyskinesias that adversely affect functional performance. Consequently, physiotherapy and exercise-based rehabilitation have become essential components of comprehensive PD management, with the goals of preserving mobility, maximizing functional independence, and improving quality of life [4].

Exercise-based rehabilitation has received increasing attention because of its potential to improve motor performance while simultaneously promoting neuroplastic adaptations within the central nervous system. Emerging evidence suggests that structured exercise enhances neural efficiency, facilitates motor learning, improves synaptic function, and promotes functional reorganization of neural pathways [5]. Numerous rehabilitation approaches have demonstrated beneficial effects in individuals with PD. Previous case reports have also described chiropractic care as a rehabilitation approach in individuals with PD, with reported improvements in gait, balance, mobility, functional independence, and postural alignment [6-8]. Treadmill-based exercise programs have been associated with improvements in gait and walking performance [9,10], while resistance training interventions have demonstrated positive effects on muscle strength, physical performance, and motor symptoms [11,12]. Similarly, task-specific training, cue-based interventions, and adaptive movement programs have shown favourable effects on mobility, motor control, and functional performance [13,14]. Other rehabilitation modalities, including Pilates, hydrotherapy, and tandem cycling, have also demonstrated improvements in balance, postural stability, endurance, and overall physical function [15-17]. Collectively, these findings support the important role of structured rehabilitation in the management of individuals with PD.

Despite substantial evidence supporting exercise-based rehabilitation, considerable variability exists in the magnitude of improvement achieved by different individuals. While some patients demonstrate marked gains in mobility and balance, others experience comparatively limited improvement following similar rehabilitation programs. This variability suggests that patient-specific characteristics may influence rehabilitation responsiveness. Among these factors, baseline disease severity is of particular clinical importance because it reflects the extent of neurological involvement, motor impairment, functional limitation, and disease progression [18].

The Modified Hoehn and Yahr stage is one of the most widely accepted methods for classifying disease severity in PD [18]. It categorizes patients according to the progression of motor disability, ranging from mild unilateral involvement to advanced disease characterized by marked postural instability and impaired independent mobility. Previous studies have demonstrated that increasing disease severity is associated with poorer balance, reduced mobility, diminished physical performance, greater functional dependence, and lower quality of life [19-21]. Consequently, individuals at different disease stages may exhibit varying capacities to respond to rehabilitation interventions and may require individualized therapeutic approaches to achieve optimal functional outcomes.

The influence of baseline disease severity on rehabilitation outcomes is particularly relevant in individuals with chronic PD, where progressive motor impairments frequently necessitate ongoing physiotherapy and long-term exercise-based management. Understanding stage-specific rehabilitation responses may assist clinicians in developing individualized treatment plans, establishing realistic therapeutic goals, and optimizing exercise prescription according to patient needs. Furthermore, identifying differences in rehabilitation responsiveness across disease stages may contribute to more efficient utilization of rehabilitation resources and improved evidence-based clinical decision-making.

Although numerous studies have reported the effectiveness of exercise interventions in PD, most investigations have focused primarily on overall treatment effects in mixed-stage populations without specifically examining whether baseline disease severity influences the magnitude of rehabilitation-related improvement [9-17]. Consequently, evidence regarding the relationship between baseline Modified Hoehn and Yahr stage and rehabilitation outcomes remains limited. To the best of our knowledge, few studies have specifically explored the influence of disease severity on rehabilitation outcomes following a structured exercise program in individuals with chronic PD. A better understanding of this relationship may help determine whether individuals at different stages of disease progression derive comparable benefits from rehabilitation and provide valuable information for stage-specific rehabilitation planning. Therefore, this secondary analysis aimed to examine the relationship between baseline Modified Hoehn and Yahr stage and changes in functional mobility and balance, assessed using the Timed Up and Go (TUG) test and Berg Balance Scale (BBS), following a structured six-week physiotherapy program in individuals with chronic PD. Changes in Modified Hoehn and Yahr stage from baseline to post-intervention were also examined descriptively as a secondary outcome.

## Materials and methods

Study design

The present study was conducted as a secondary analysis of data derived from a previously completed single-group pretest-posttest quasi-experimental study that evaluated the effects of a structured physiotherapy rehabilitation program in individuals with chronic PD [22]. While the parent study primarily investigated the effectiveness of the rehabilitation program on functional mobility, balance, and disease severity, the objective of the present secondary analysis was to examine the relationship between baseline disease severity and rehabilitation outcomes.

Ethical considerations

Ethical approval for the parent study was obtained from the Institutional Ethics Committee of Krishna Vishwa Vidyapeeth (Deemed to be University), Karad (approval no. KVV/IEC/10/2025) prior to commencement of the study. All study procedures were conducted in accordance with the ethical principles of the Declaration of Helsinki, and written informed consent was obtained from all participants before enrolment in the parent study [22].

The present investigation was conducted as a secondary analysis of data obtained from the previously approved study. No additional participant recruitment, intervention, or data collection was undertaken for the present analysis.

Study setting and participants

The parent study was conducted between November 5, 2025, and March 31, 2026, at the Neurosciences Outpatient Department of Krishna College of Physiotherapy, Karad.

A total of 20 individuals with clinically diagnosed chronic PD were recruited using a consecutive sampling method. The study population comprised 10 male patients and 10 female patients aged between 50 and 70 years. All participants who completed both baseline and post-intervention assessments in the parent study were included in the present secondary analysis.

For the purpose of this study, chronic PD was operationally defined as a clinically confirmed diagnosis of PD associated with persistent and progressive motor impairments, functional limitations, and the requirement for ongoing physiotherapy rehabilitation.

Eligibility criteria

Participants with clinically confirmed PD classified within Modified Hoehn and Yahr stages II-V were eligible for inclusion. Disease severity was classified using the Modified Hoehn and Yahr stage, which categorizes PD according to the progression of motor impairment and functional disability.

Participants were required to be medically stable, capable of understanding and following simple verbal instructions, and able to stand and ambulate independently or with assistance.

Individuals were excluded if they presented with severe cognitive impairment or dementia, neurological disorders other than PD affecting mobility, severe musculoskeletal conditions limiting balance or lower-limb function, recent fractures or orthopedic surgery within the previous six months, severe visual or vestibular impairments affecting postural control, or medical conditions contraindicating moderate physical exercise.

Intervention

All participants underwent a structured, stage-based physiotherapy rehabilitation program for six weeks, with five supervised sessions per week and each session lasting approximately 45 minutes. The program was designed to address the major motor and functional impairments associated with chronic PD, including functional mobility, gait, balance, postural control, muscle strength, flexibility, coordination, and task-specific functional performance.

Exercise selection and progression were individualized according to the participant's modified Hoehn and Yahr stage, clinical presentation, functional capacity, movement quality, safety, and tolerance. The intervention incorporated active range-of-motion exercises, flexibility and stretching exercises, strengthening exercises, aerobic conditioning, gait and functional mobility training, balance and postural control exercises, coordination training, trunk stabilization, transfer training, task-specific activities, and external cueing strategies when clinically appropriate.

For participants with milder disease, the program emphasized active mobility, flexibility, strengthening, aerobic conditioning, postural correction, functional task practice, gait training, and movement retraining. For participants with more advanced disease, the program was modified to emphasize assisted gait and transfer training, supported balance activities, assisted or gravity-eliminated strengthening, trunk mobility, respiratory exercises, bed mobility, positioning, and caregiver-assisted movement as required. Participants in intermediate stages received progressively challenging balance, gait, coordination, strengthening, trunk stabilization, and functional task training according to their functional capacity.

Exercise dosage was adjusted according to clinical response and patient tolerance. Progression was achieved by increasing repetitions, exercise duration, resistance, gait complexity, balance challenge, coordination demands, or task-specific functional demands when participants demonstrated adequate movement quality, stable balance, appropriate exercise tolerance, and the ability to perform the prescribed activity safely. Exercise intensity was guided by patient tolerance and rating of perceived exertion (RPE), where applicable. Exercises were reduced or modified in the presence of excessive fatigue, dizziness, loss of postural control, or other adverse symptoms.

The intervention included supervised delivery by qualified physiotherapists, with attendance recorded for each session to monitor adherence. The stage-specific exercise components, their corresponding dosage, and complete intervention protocol was also reported in the parent study [22].

Outcome measures

Timed Up and Go (TUG) test

Functional mobility was assessed using the TUG test. Participants were instructed to rise from a chair, walk three meters, turn around, return to the chair, and sit down. The total time required to complete the task was recorded in seconds. Lower scores indicated better functional mobility.

Berg Balance Scale (BBS)

Balance performance was evaluated using the BBS, a 14-item functional balance assessment with a maximum score of 56. Higher scores indicated better balance and postural control.

Modified Hoehn and Yahr stage

Disease severity was assessed using the Modified Hoehn and Yahr stage, which classifies PD according to the progression of motor impairment and functional disability.

Data collection

Outcome data were retrieved from the database of the parent study. Assessments were performed at baseline and immediately after completion of the six-week rehabilitation program using standardized testing procedures.

For the present secondary analysis, baseline modified Hoehn and Yahr stage was considered the primary indicator of disease severity. Rehabilitation outcomes were quantified using individual change scores calculated from baseline and post-intervention measurements. For the TUG test, improvement was calculated as the baseline score minus the post-intervention score because lower TUG values indicate better functional mobility. For the BBS, improvement was calculated as the post-intervention score minus the baseline score because higher BBS values indicate better balance performance. Thus, positive change scores represented improvement for both outcome measures, with larger positive values indicating greater improvement.

Statistical analysis

Statistical analysis was performed using IBM SPSS Statistics for Windows, Version 31 (Released 2025; IBM Corp., Armonk, New York, United States). Continuous variables were summarized using mean ± standard deviation (SD) and, where appropriate, median with interquartile range (IQR). Categorical variables were presented as frequencies and percentages.

For the present secondary analysis, change scores were calculated for each functional outcome as described above. The distribution of the change scores was examined using descriptive statistics and the Shapiro-Wilk test. Change-score variability was summarized using the mean and SD, with median and IQR reported where appropriate.

The relationship between baseline disease severity and rehabilitation outcomes was examined using Spearman's rank correlation coefficient (rs). Spearman correlation was selected because baseline modified Hoehn and Yahr stage is an ordinal measure and the analysis involved a small sample. Correlation analyses examined the associations between baseline modified Hoehn and Yahr stage and change scores for the TUG test and BBS. Because this was a secondary analysis, these correlation analyses were considered exploratory and were not interpreted as evidence of a causal relationship.

Changes in modified Hoehn and Yahr stage between baseline and post-intervention assessments were analyzed using the Wilcoxon signed-rank test.

Potential clinical factors that could influence rehabilitation outcomes, including age, baseline disease severity, disease duration, medication status, and individual functional capacity, were considered as potential confounding factors during interpretation. However, multivariable adjustment was not performed because of the small sample size and the exploratory nature of the secondary analysis. These factors were therefore considered as potential sources of residual confounding and are acknowledged as limitations.

Correlation coefficients were interpreted as weak (0.00-0.39), moderate (0.40-0.69), and strong (0.70-1.00). Statistical significance was established at a p-value of <0.05.

## Results

A total of 20 participants with chronic PD were included in the study. The study population consisted of 10 male patients (50%) and 10 female patients (50%). The mean age of the participants was 60.4 ± 5.8 years, with an age range of 50-70 years. The demographic characteristics of the participants are presented in Table 1.

**Table 1:** Demographic characteristics of the participants (n=20) SD, standard deviation; n, number of participants.

Variable	Value
Age (years), mean ± SD	60.4 ± 5.8
Male, n (%)	10 (50%)
Female, n (%)	10 (50%)

No participants were classified within Modified Hoehn and Yahr stage 1 or stage 1.5 at baseline. Among the study population, four participants (20%) were classified as stage 2, six participants (30%) as stage 2.5, five participants (25%) as stage 3, three participants (15%) as stage 4, and two participants (10%) as stage 5. The distribution of participants according to baseline disease severity is presented in Table 2.

**Table 2:** Distribution of participants according to baseline Modified Hoehn and Yahr stage

Modified Hoehn and Yahr Stage	Number of Participants (n)	Percentage (%)
Stage 2	4	20
Stage 2.5	6	30
Stage 3	5	25
Stage 4	3	15
Stage 5	2	10

Rehabilitation-related change scores were calculated from baseline and post-intervention measurements. For the TUG test, improvement was calculated as the baseline score minus the post-intervention score, whereas for the BBS, improvement was calculated as the post-intervention score minus the baseline score. Thus, positive change scores represented improvement for both outcomes.

The mean improvement in TUG performance was 4.4 ± 2.51 seconds, while the mean improvement in BBS score was 8.3 ± 4.52 points. The baseline, post-intervention, and change-score characteristics are presented in Table 3.

**Table 3:** Baseline, post-intervention, and change scores for functional mobility and balance Positive change scores indicate improvement. TUG change = baseline score − post-intervention score; BBS change = post-intervention score − baseline score. SD, standard deviation; TUG, Timed Up and Go; BBS, Berg Balance Scale.

Outcome measure	Baseline, mean ± SD	Post-intervention, mean ± SD	Mean change ± SD
TUG (seconds)	18.6 ± 3.2	14.2 ± 2.8	4.4 ± 2.51
BBS (points)	34.5 ± 5.1	42.8 ± 4.7	8.3 ± 4.52

The relationship between baseline disease severity and rehabilitation outcomes was examined using Spearman's rank correlation analysis. A statistically significant moderate positive correlation was observed between baseline Modified Hoehn and Yahr stage and improvement in TUG performance (rₛ=0.53, p=0.015).

A weak positive correlation was observed between baseline Modified Hoehn and Yahr stage and improvement in BBS scores (rₛ=0.30, p=0.207); however, this association was not statistically significant. The correlation analysis results are presented in Table 4.

**Table 4:** Correlation between baseline disease severity and rehabilitation-related change scores H&Y, Hoehn and Yahr; TUG, Timed Up and Go; BBS, Berg Balance Scale; r_s_, Spearman's rank correlation coefficient. *Statistically significant at p<0.05.

Correlation Analysis	Spearman's r_s_	p-value
Baseline H&Y Stage vs TUG Improvement	0.53	0.015*
Baseline H&Y Stage vs BBS Improvement	0.30	0.207

Changes in Modified Hoehn and Yahr stage following rehabilitation were evaluated using the Wilcoxon signed-rank test. The median Modified Hoehn and Yahr stage decreased from 2.75 (IQR: 2.50-3.25) at baseline to 2.00 (IQR: 2.00-2.63) following completion of the rehabilitation program. The reduction in Modified Hoehn and Yahr stage was statistically significant (W=3.5, p<0.001).

Overall, baseline Modified Hoehn and Yahr stage was significantly associated with improvement in functional mobility as measured by the TUG test (rₛ=0.53, p=0.015), whereas no statistically significant association was observed between baseline disease severity and improvement in BBS scores (rₛ=0.30, p=0.207). Furthermore, a statistically significant reduction in Modified Hoehn and Yahr stage was observed following completion of the rehabilitation program, with the median stage decreasing from 2.75 (IQR: 2.50-3.25) at baseline to 2.00 (IQR: 2.00-2.63) post-intervention (W=3.5, p<0.001).

## Discussion

The present secondary analysis extends the findings of a previously published interventional study by examining the influence of baseline disease severity on rehabilitation outcomes following a structured physiotherapy rehabilitation program in individuals with chronic PD [22]. The findings demonstrated a statistically significant moderate positive association between baseline Modified Hoehn and Yahr stage and improvement in functional mobility as measured by the TUG test, suggesting that participants with greater baseline disease severity tended to demonstrate larger improvements in functional mobility following rehabilitation. In contrast, no statistically significant association was observed between baseline disease severity and improvement in balance performance as measured by the BBS. Furthermore, a statistically significant reduction in Modified Hoehn and Yahr stage was observed following completion of the rehabilitation program, which may reflect improvement in overall motor function and disease-related disability.

PD is a progressive neurodegenerative disorder characterized by impairments in gait, mobility, postural control, and functional independence resulting from degeneration of dopaminergic neurons and dysfunction of basal ganglia circuits [1,2]. As disease severity advances, individuals commonly experience greater limitations in mobility, balance, and daily activities, leading to increased disability and reduced participation in social and functional roles [5]. Understanding whether baseline disease severity is associated with rehabilitation responsiveness is therefore important for optimizing clinical decision-making and individualized treatment planning.

A major finding of the present study was the significant positive correlation between baseline Modified Hoehn and Yahr stage and improvement in TUG performance. Participants with more advanced baseline disease severity tended to demonstrate greater improvements in functional mobility following rehabilitation. Although greater disease severity is generally associated with poorer baseline functional performance, individuals with greater initial impairment may have a larger scope for measurable improvement following rehabilitation. Consequently, greater absolute changes may be observed among individuals with more pronounced baseline functional limitations. Previous evidence has suggested that baseline functional impairment may influence the magnitude of change observed following exercise interventions, with individuals presenting greater mobility limitations potentially demonstrating larger improvements in mobility-related outcomes [20,21]. However, this interpretation should be considered cautiously because the present analysis did not determine whether baseline disease severity independently predicted improvement after accounting for other clinical characteristics.

The mobility-related findings are consistent with previous research supporting exercise-based rehabilitation in PD. Treadmill-based interventions have demonstrated beneficial effects on gait performance, walking ability, and mobility outcomes [11]. Similarly, high-intensity exercise programs have been associated with improvements in motor symptoms and physical function [12]. Progressive resistance training has also been shown to enhance strength, physical performance, and functional mobility [13,14]. In addition, task-specific and cue-based training approaches have demonstrated positive effects on movement execution and mobility-related activities [15,16]. Collectively, these findings support the potential role of structured rehabilitation programs in improving mobility across different stages of PD.

Several mechanisms may explain the observed improvements in functional mobility. The rehabilitation program incorporated gait training, balance retraining, strengthening exercises, flexibility activities, postural correction, coordination training, transfer practice, and task-oriented functional exercises tailored according to disease severity and individual functional capacity. Repetitive task-specific practice and progressive exercise training may enhance motor learning, improve neuromuscular coordination, and promote more efficient movement strategies. Exercise-induced neuroplasticity has been proposed as an important mechanism underlying rehabilitation-related improvements in PD, with evidence suggesting that structured exercise may facilitate adaptive neural reorganization, enhance synaptic efficiency, and strengthen compensatory motor pathways [3]. These adaptations may contribute to improved mobility and functional performance despite the progressive nature of the disease.

In contrast to functional mobility, baseline disease severity was not significantly associated with improvement in BBS scores. Although balance performance improved following rehabilitation, the magnitude of improvement did not appear to vary significantly according to baseline Modified Hoehn and Yahr stage. This finding suggests that balance-related improvements may occur across a broad range of disease severity. However, the absence of a statistically significant association should not be interpreted as evidence that disease severity has no influence on balance responsiveness, particularly given the small sample size and limited statistical power of the present analysis.

The absence of a significant relationship between disease severity and balance improvement may be explained by the multifactorial nature of postural control deficits in PD. Balance dysfunction arises from complex interactions among impaired sensory integration, altered postural responses, bradykinesia, rigidity, reduced automaticity of movement, and deficits in motor planning [1]. Because balance performance is influenced by multiple physiological and functional systems, improvements in balance may not necessarily follow the same severity-dependent pattern observed for functional mobility. Previous studies have reported significant improvements in balance and postural stability following Pilates-based rehabilitation programs [17], hydrotherapy interventions [18], and cycling-based exercise programs [19]. Therefore, the present findings suggest that balance-oriented rehabilitation may provide benefits across different levels of disease severity, although larger studies are required to determine whether the magnitude of these benefits differs between disease stages.

Another important finding was the statistically significant reduction in Modified Hoehn and Yahr stage following rehabilitation. Although the Modified Hoehn and Yahr stage is primarily used to classify disease severity rather than to assess short-term treatment responsiveness [5], the observed reduction in stage may reflect improvements in overall motor function and disease-related disability following rehabilitation. This finding supports the importance of physiotherapy and neurorehabilitation as components of comprehensive PD management [10]. While pharmacological treatment remains central to symptomatic management, rehabilitation interventions play an important role in maintaining mobility, enhancing postural control, reducing functional disability, and preserving independence throughout the disease course [1,10]. Nevertheless, changes in a staging measure over a relatively short intervention period should be interpreted cautiously, as the Modified Hoehn and Yahr scale was not specifically designed to quantify short-term rehabilitation-related change.

The present study contributes to the existing literature by examining the relationship between baseline disease severity and rehabilitation-related change rather than focusing solely on the overall effectiveness of the intervention. Most previous investigations have evaluated whether exercise interventions improve gait, balance, mobility, and motor performance [11-19]. Comparatively fewer studies have examined whether baseline disease severity is associated with the magnitude of rehabilitation-related improvement. The present findings suggest that individuals with greater baseline disease severity may still achieve meaningful improvements in functional mobility following structured physiotherapy rehabilitation. However, because the present analysis was observational with respect to the association between baseline severity and change scores, the observed relationship should be interpreted as an association rather than evidence that greater disease severity directly causes greater rehabilitation-related improvement.

Potential confounding should also be considered when interpreting this association. Factors such as age, sex, disease duration, medication status and timing of medication administration, baseline functional capacity, cognitive status, comorbidities, and adherence to the rehabilitation program may influence both baseline disease severity and the magnitude of functional improvement. For example, differences in medication response or medication timing during outcome assessment could influence motor performance independently of the rehabilitation intervention. Similarly, differences in disease duration, baseline mobility, cognitive function, or individual exercise tolerance may affect the capacity to respond to rehabilitation. These factors were not examined as independent predictors in the present secondary analysis, and no multivariable adjustment was performed. Consequently, residual confounding cannot be excluded, and the observed association between baseline Modified Hoehn and Yahr stage and TUG improvement may be partly influenced by unmeasured or uncontrolled participant characteristics.

An additional interpretive consideration is the individualized nature of the rehabilitation program. The intervention was tailored according to disease severity and individual functional capacity; therefore, exercise selection, task difficulty, intensity, and progression may have differed among participants with different baseline severity levels. Consequently, the observed association between baseline Modified Hoehn and Yahr stage and improvement in TUG performance may partly reflect differences in the rehabilitation exposure rather than an independent effect of baseline disease severity. Because the present secondary analysis did not quantify or adjust for differences in exercise selection, intensity, or progression, the independent contribution of baseline disease severity to rehabilitation-related improvement cannot be established.

The use of change scores also warrants consideration when interpreting the correlation findings. Change scores are useful for quantifying rehabilitation-related improvement; however, the magnitude of change may be influenced by baseline performance, measurement variability, and regression toward the mean. Participants with poorer baseline performance may have greater potential for measurable improvement, which could contribute to the observed association between baseline disease severity and TUG change. Although the present analysis quantified variability in TUG and BBS change scores and examined their associations with baseline Modified Hoehn and Yahr stage, it was not designed to determine independent predictors of treatment response. Future studies should therefore consider analytical approaches that account for baseline outcome values and relevant clinical characteristics when investigating determinants of rehabilitation responsiveness.

From a clinical perspective, these findings highlight the importance of providing structured rehabilitation across different stages of PD. Clinicians may sometimes anticipate limited rehabilitation potential in individuals with advanced disease; however, the present findings suggest that meaningful mobility-related improvements may still be achieved among individuals with greater baseline disease severity. Rehabilitation programs should therefore be individualized according to patient needs, functional abilities, safety, and rehabilitation goals rather than being restricted solely on the basis of disease stage. However, these findings should not be interpreted as demonstrating that advanced disease independently predicts a greater rehabilitation response, given the potential influence of baseline characteristics, confounding factors, and differences in individualized rehabilitation exposure.

A major strength of the present study is that it extends the findings of the parent interventional study by exploring rehabilitation responsiveness according to baseline disease severity. Rather than evaluating the effectiveness of the intervention alone, this secondary analysis provides additional information regarding the relationship between baseline disease severity and functional change following rehabilitation. The use of standardized clinical outcome measures, including the TUG, BBS, and Modified Hoehn and Yahr stage, also provides clinically interpretable measures of mobility, balance, and disease-related functional status.

Several limitations should be acknowledged. First, the small sample size (n=20) limited statistical power, particularly for detecting associations involving balance outcomes, made comparisons across individual Modified Hoehn and Yahr stages potentially unstable, and restricted the generalizability of the findings. The small sample also increases the risk of type II error and over-interpretation of the observed correlation findings. Second, the investigation was conducted as a secondary analysis of a single-group pretest-posttest study without a control group, limiting the ability to establish causal relationships between rehabilitation and the observed changes or between baseline disease severity and rehabilitation responsiveness. Third, disease severity was assessed using the Modified Hoehn and Yahr stage, which primarily reflects motor disability and may not fully capture non-motor manifestations of PD. Fourth, the relatively short intervention period limits conclusions regarding the long-term sustainability of rehabilitation-related improvements. Additionally, potential confounding factors, including medication status and timing, disease duration, baseline functional capacity, cognitive status, comorbidities, and adherence, were not included in adjusted analyses. Therefore, the observed relationship between baseline disease severity and TUG improvement cannot be considered an independent effect of disease severity. Furthermore, because the rehabilitation program was individualized according to disease severity and functional capacity, differences in exercise selection, intensity, task difficulty, or progression may have contributed to differences in observed outcomes. This limits the ability to separate the influence of baseline disease severity from the influence of differences in rehabilitation exposure. Finally, the use of change scores may introduce the influence of baseline performance and regression toward the mean, which should be considered when interpreting the observed correlations.

Despite these limitations, the present study provides clinically relevant insights into the relationship between baseline disease severity and rehabilitation outcomes in individuals with chronic PD. Greater baseline disease severity was associated with larger improvements in functional mobility, whereas balance-related improvements were not significantly associated with baseline disease severity. These findings should be considered exploratory and hypothesis-generating rather than definitive evidence of a severity-dependent rehabilitation response. The findings support the potential value of structured physiotherapy rehabilitation across different levels of disease severity, while emphasizing that rehabilitation response may be influenced by multiple patient- and intervention-related factors. Future prospective studies with larger samples, appropriate control groups, longer follow-up periods, standardized or clearly quantified intervention protocols, and prespecified multivariable analyses are warranted to determine whether baseline disease severity independently influences rehabilitation responsiveness after accounting for relevant demographic, clinical, medication-related, functional, and intervention-related factors.

## Conclusions

The present secondary analysis suggests that baseline disease severity may influence rehabilitation outcomes in individuals with chronic PD undergoing structured physiotherapy rehabilitation. Greater baseline Modified Hoehn and Yahr stage was significantly associated with greater improvements in functional mobility, whereas balance-related improvements were observed across disease stages without a significant association with baseline disease severity. A significant reduction in Modified Hoehn and Yahr stage was also observed following rehabilitation, which may reflect an improvement in overall motor function and disease-related disability. These findings highlight the importance of individualized physiotherapy rehabilitation tailored to disease severity and suggest that advanced PD should not be considered a barrier to participation in structured rehabilitation programs. Further prospective studies with larger sample sizes, controlled study designs, and longer follow-up periods are warranted to confirm these findings and further clarify the influence of baseline disease severity on rehabilitation responsiveness.
